# Eccentric Hamstring Force Asymmetry After Anterior Cruciate Ligament Reconstruction: A Cross-Sectional Comparison of Independent 12- and 24-Month Postoperative Cohorts

**DOI:** 10.3390/diagnostics16183042

**Published:** 2026-09-19

**Authors:** Georgios Kakavas, Nikolaos Malliaropoulos, Robert Prill, Florian Forelli

**Affiliations:** 1 Fysiotek Spine & Sports Lab, 17562 Athens, Greece; 2 Sports & Exercise Medicine Clinic, Barts Health NHS Trust, London E1 1BB, UK; 3 Center for Sports & Exercise Medicine, Queen Mary University of London, London E1 4NS, UK; 4 Center of Orthopaedics and Traumatology, University Hospital Brandenburg/Havel, Brandenburg Medical School Theodor Fontane, 14770 Brandenburg an der Havel, Germany; 5 Haute-Ecole Arc Santé, HES-SO University of Applied Sciences and Arts Western Switzerland, 2800 Delémont, Switzerland; 6 Orthopaedic Surgery Department, Clinic of Domont, Ramsay Healthcare, @OrthoLab, 95330 Domont, France; 7 Société Française des Masseurs-Kinésithérapeutes du Sport Lab, 93380 Pierrefitte-sur-Seine, France

**Keywords:** anterior cruciate ligament reconstruction, hamstrings, eccentric strength, Nordic hamstring exercise, limb symmetry index, postoperative assessment

## Abstract

**Background/Objectives:** Prior NordBord studies have documented eccentric knee-flexor asymmetry during the first postoperative year and at later follow-up after hamstring-autograft ACLR. This study directly compared relative and Newton-based eccentric hamstring deficits between independent male-athlete cohorts tested with the same single-center protocol approximately 12 and 24 months after ACLR. **Methods:** In this single-center cross-sectional comparative study, 60 male athletes were assessed once in an independent 12-month cohort (n = 30) or 24-month cohort (n = 30) after unilateral primary ACLR. Bilateral eccentric peak force (PF) and mean force (MF) during the Nordic hamstring exercise were recorded with a NordBord device at 50 Hz. For PF, the highest value across three maximal trials was retained independently for each limb, so the retained involved- and uninvolved-limb values could originate from different trials. The primary outcome was PF limb symmetry index (LSI; involved/uninvolved × 100); secondary outcomes included MF LSI, bilateral PF values, and PF LSI < 90%. **Results:** PF LSI was 84.6 ± 9.3% in the 12-month cohort and 93.5 ± 6.8% in the 24-month cohort (mean difference 8.9 percentage points, 95% CI 4.7–13.1; *p* < 0.001; d = 1.09). Involved-limb PF was 32.5 N higher in the 24-month cohort (95% CI 6.8–58.2; *p* = 0.014; d = 0.65), whereas uninvolved-limb PF was similar between cohorts (mean difference 2.5 N, 95% CI −21.9 to 26.9; *p* = 0.838; d = 0.05). PF LSI was <90% in 21/30 athletes (70.0%; 95% CI 52.1–83.3%) in the 12-month cohort and 7/30 (23.3%; 95% CI 11.8–40.9%) in the 24-month cohort (χ^2^ = 13.13; *p* < 0.001). **Conclusions:** The smaller eccentric hamstring asymmetry observed in the independent 24-month cohort was driven predominantly by higher involved-limb force rather than by a difference in uninvolved-limb force, yet nearly one quarter of that cohort remained below the 90% LSI benchmark. Postoperative time alone should therefore not substitute for late-stage objective eccentric knee-flexor assessment.

## 1. Introduction

Return to sport (RTS) and restoration of lower-limb function after anterior cruciate ligament reconstruction (ACLR) are heterogeneous, and residual strength deficits may persist despite completion of conventional rehabilitation [1,2,3,4,5]. Contemporary rehabilitation and RTS frameworks therefore emphasize criterion-based, multidimensional assessment rather than time alone [3,4,5,6]. The period around 9–12 months is a clinically important late-rehabilitation and RTS decision window, whereas assessment around 24 months provides a later postoperative stage at which persistent knee-flexor impairment may still be relevant [5,6,7,8]. Direct comparison of these stages can therefore help determine whether clinically detectable eccentric deficits remain after the time at which many athletes have resumed sport-related activity [6,8,9].

The hamstrings contribute to dynamic knee control and restraint of anterior tibial translation [10,11]. Interpretation of knee-flexor performance after ACLR is particularly important when semitendinosus and gracilis tendons have been harvested, because altered recruitment, variable tendon regeneration, and persistent musculotendinous morphological changes have been reported after hamstring-autograft reconstruction [12,13,14,15]. Isokinetic studies also show that graft choice, contraction mode, and joint angle influence the magnitude of postoperative knee-flexor deficits [16,17,18,19,20,21,22,23]. NordBord studies by Högberg and colleagues have demonstrated eccentric knee-flexor asymmetry during the first postoperative year and at two- and five-year follow-up after hamstring-autograft ACLR [8,24].

Portable Nordic hamstring devices provide a clinically accessible measure of bilateral eccentric knee-flexor force, with acceptable reliability for peak-force assessment, although interlimb asymmetry is more variable and method-dependent [25,26,27]. Limb symmetry indices (LSIs) are widely used after ACLR, but they may overestimate recovery if the uninvolved limb is also deconditioned; reporting bilateral absolute force values alongside LSI can therefore add complementary information [5,6,28]. Reduced force output is also mechanistically nonspecific and may reflect donor site changes, muscle atrophy, pain, apprehension, insufficient loading, or altered neural activation rather than a single process such as arthrogenic muscle inhibition (AMI) [14,15,29,30,31,32,33]. Molecular biomarkers have been investigated after ACL injury and reconstruction, but they are not validated markers of eccentric hamstring weakness or RTS readiness [34,35,36,37].

Accordingly, the present study was not designed to establish for the first time that NordBord asymmetry can persist after ACLR, as this has already been shown [8,24]. Its added value was to compare independent cohorts at two clinically distinct postoperative stages under the same single-center testing protocol while reporting PF LSI, bilateral PF values, and the proportion below a commonly used 90% LSI benchmark. This bilateral reporting also allows the between-cohort contrast to be localized to the involved limb, the uninvolved limb, or both rather than relying on LSI alone [28].

The primary aim was therefore to compare PF LSI between independent cohorts of male athletes assessed at approximately 12 and 24 months after ACLR. Secondary aims were to compare MF LSI and bilateral PF values, describe the proportion of athletes with PF LSI < 90%, and determine whether the between-cohort PF contrast was primarily attributable to the involved or uninvolved limb. We hypothesized that the 24-month cohort would demonstrate greater PF symmetry than the 12-month cohort, without inferring within-person recovery over time.

## 2. Materials and Methods

### 2.1. Study Design and Ethics

This single-center cross-sectional comparative study included two independent cohorts of male athletes who had undergone unilateral primary ACLR. One cohort was assessed at approximately 12 months after surgery and the second at approximately 24 months. Each participant contributed data at one postoperative time point only; consequently, the study was designed to quantify between-cohort differences and not longitudinal recovery within the same individuals. All assessments were conducted at the same sports-medicine facility using a standardized testing sequence and the same eccentric knee-flexor device. Reporting was structured according to the Strengthening the Reporting of Observational Studies in Epidemiology (STROBE) recommendations [38].

The study received ethical approval from the University of Athens/Department of Physical Education and Sports Science Ethics Board (IRB reference 28564/23; approved 1 October 2022) and was conducted in accordance with the Declaration of Helsinki. All participants provided written informed consent before participation.

### 2.2. Participants

Sixty male athletes were included, with 30 participants in the independent 12-month cohort and 30 in the independent 24-month cohort. Eligibility criteria were: (1) age 18–40 years; (2) unilateral primary ACLR using either a hamstring tendon autograft (semitendinosus/gracilis) or bone–patellar tendon–bone (BPTB) autograft; (3) full weight-bearing and participation in structured postoperative rehabilitation for at least six months before assessment; and (4) ability to perform maximal Nordic hamstring testing. Exclusion criteria were revision ACLR, contralateral lower-extremity pathology, a concurrent lower-limb injury at the time of testing, intra-articular corticosteroid injection within the preceding three months, a neurological disorder affecting lower-limb motor control, or inability to perform a maximal Nordic hamstring effort because of pain or apprehension. The retained study records do not preserve the recruitment sequence or a prospective screening log; therefore, whether recruitment was consecutive or based on convenience cannot be confirmed, and the total number screened and detailed reasons for exclusion cannot be reconstructed reliably.

Participant characteristics recorded for descriptive comparison included age, height, body mass, postoperative stage, graft type, and Tegner Activity Score. Hamstring tendon autograft accounted for 70% of reconstructions in each cohort and BPTB autograft for 30%, so graft-type proportions were identical between cohorts. Rehabilitation was not experimentally standardized by the study; eligibility required only that participants had engaged in structured rehabilitation for at least six months. A formal a priori sample-size calculation was not documented in the retained study records; a literature-informed benchmark calculation is therefore reported transparently in the Statistical Analysis section.

### 2.3. Eccentric Hamstring Force Assessment

Eccentric knee-flexor force was assessed bilaterally using a NordBord dynamometer (VALD Performance, Newstead, Brisbane, QLD, Australia). The device records uniaxial force independently from the right and left ankle restraints at a sampling frequency of 50 Hz during the Nordic hamstring exercise, enabling simultaneous bilateral force acquisition. Device-based Nordic testing has been used extensively to quantify eccentric knee-flexor capacity and interlimb asymmetry [25,26]. All assessments were supervised by a single trained examiner who was not involved in the participants’ rehabilitation, and the same standardized protocol was applied to both postoperative cohorts.

Before maximal testing, participants completed a five-minute stationary cycle warm-up at 80–100 revolutions per minute. They then performed three submaximal Nordic hamstring repetitions for familiarization, followed by two minutes of rest. The maximal test consisted of three maximal-effort Nordic hamstring trials separated by one minute of recovery. For PF, the highest value across the three trials was selected separately for each limb. Consequently, the retained involved- and uninvolved-limb PF values could originate from different trials. This limb-specific maximum convention is consistent with published NordBord protocols that retain maximal peak force across repeated efforts [25].

Force data were exported from the manufacturer’s software for analysis. Peak force (PF, N) was defined as the highest force recorded after the onset of the eccentric movement. Mean force (MF, N) represented the mean force recorded between movement onset and the PF event. PF was selected as the primary force variable because it provides a direct measure of maximal eccentric output, whereas MF was retained as a secondary descriptor of force production across the eccentric phase. Both outcomes were analyzed separately for the involved and uninvolved limbs.

The primary study outcome was the PF limb symmetry index (LSI), calculated as (involved-limb PF/uninvolved-limb PF) × 100. The same equation was applied to MF to obtain MF LSI. Values below 100% indicate lower force in the involved limb relative to the contralateral limb. Involved- and uninvolved-limb PF values were also retained and reported separately to clarify the limb contribution to between-cohort differences. The proportion of participants with PF LSI < 90% was reported as a descriptive secondary outcome. The 90% value was used as a clinical benchmark because this threshold is commonly employed within multifactorial ACLR strength and functional test batteries [5,6,28]; it was not considered a validated NordBord-specific RTS cut-off and was not used to infer readiness for unrestricted sport. The experimental sequence, derived variables, and interpretive boundaries are summarized in Table 1.

### 2.4. Statistical Analysis

Continuous variables are presented as mean ± standard deviation (SD), and categorical variables as number and percentage. Distributional assumptions for continuous outcomes were examined using the Shapiro–Wilk test. The primary between-cohort comparison was PF LSI. Other between-cohort comparisons included involved-limb PF, uninvolved-limb PF, involved-limb MF, and MF LSI. Independent-samples *t*-tests were used for continuous between-cohort comparisons, with mean differences, 95% confidence intervals (CIs), and Cohen’s d reported.

The proportions of athletes with PF LSI < 90% in the two independent postoperative cohorts were compared using Pearson’s chi-square test, and Wilson 95% CIs were calculated for cohort-specific proportions. Statistical significance was set at *p* < 0.05. The PF LSI comparison was the primary analysis; other comparisons were secondary and interpreted using effect sizes and CIs alongside nominal *p* values, without multiplicity adjustment. Exploratory summary-level comparisons were also performed for Tegner Activity Score and uninvolved-limb PF. A formal a priori sample-size calculation was not documented in the retained study records. Prior NordBord ACLR studies demonstrated persistent LSI deficits but did not provide a directly transferable standardized effect for an independent 12-versus-24-month cohort comparison [8,24]. Because no directly transferable standardized effect was available for the present independent 12-versus-24-month comparison, we used the conventional large standardized effect of Cohen’s d = 0.80 as a transparent benchmark. Using G*Power 3.1 for a two-sided independent-samples *t*-test with α = 0.05, 80% power, and 1:1 allocation, 26 participants per group (52 total) were required; the available sample of 30 participants per group exceeded this benchmark. A sensitivity calculation with the available sample indicated 80% power to detect an effect of approximately d = 0.74. This calculation was contextual and was not used prospectively to determine recruitment. Analyses were performed using SPSS version 27 (IBM Corp., Armonk, NY, USA).

## 3. Results

### 3.1. Participant Characteristics

Participant characteristics are shown in Table 2. Age, height, and body mass were descriptively similar between cohorts, and graft-type proportions were identical (70% hamstring tendon autograft and 30% BPTB in each cohort). Tegner Activity Score was 6.2 ± 1.1 in the 12-month cohort and 6.8 ± 0.9 in the 24-month cohort. An exploratory summary-level comparison indicated a mean difference of 0.6 points (95% CI 0.08–1.12; *p* = 0.024; d = 0.60). Because participant-level data were unavailable for covariate-adjusted analysis, the possibility that activity level partly contributes to the unadjusted force differences cannot be excluded.

### 3.2. Bilateral Eccentric Hamstring Force

Descriptive bilateral force outcomes are presented in Table 3. In the 12-month cohort, mean PF was 286.4 ± 52.1 N in the involved limb and 338.7 ± 48.6 N in the uninvolved limb, with a mean PF LSI of 84.6 ± 9.3%. In the 24-month cohort, mean PF was 318.9 ± 47.3 N and 341.2 ± 45.8 N, respectively, with a mean PF LSI of 93.5 ± 6.8%. Thus, the between-cohort PF contrast was concentrated in the involved limb, whereas PF in the uninvolved limb was nearly identical.

### 3.3. Between-Cohort Comparisons

**Figure 1:**
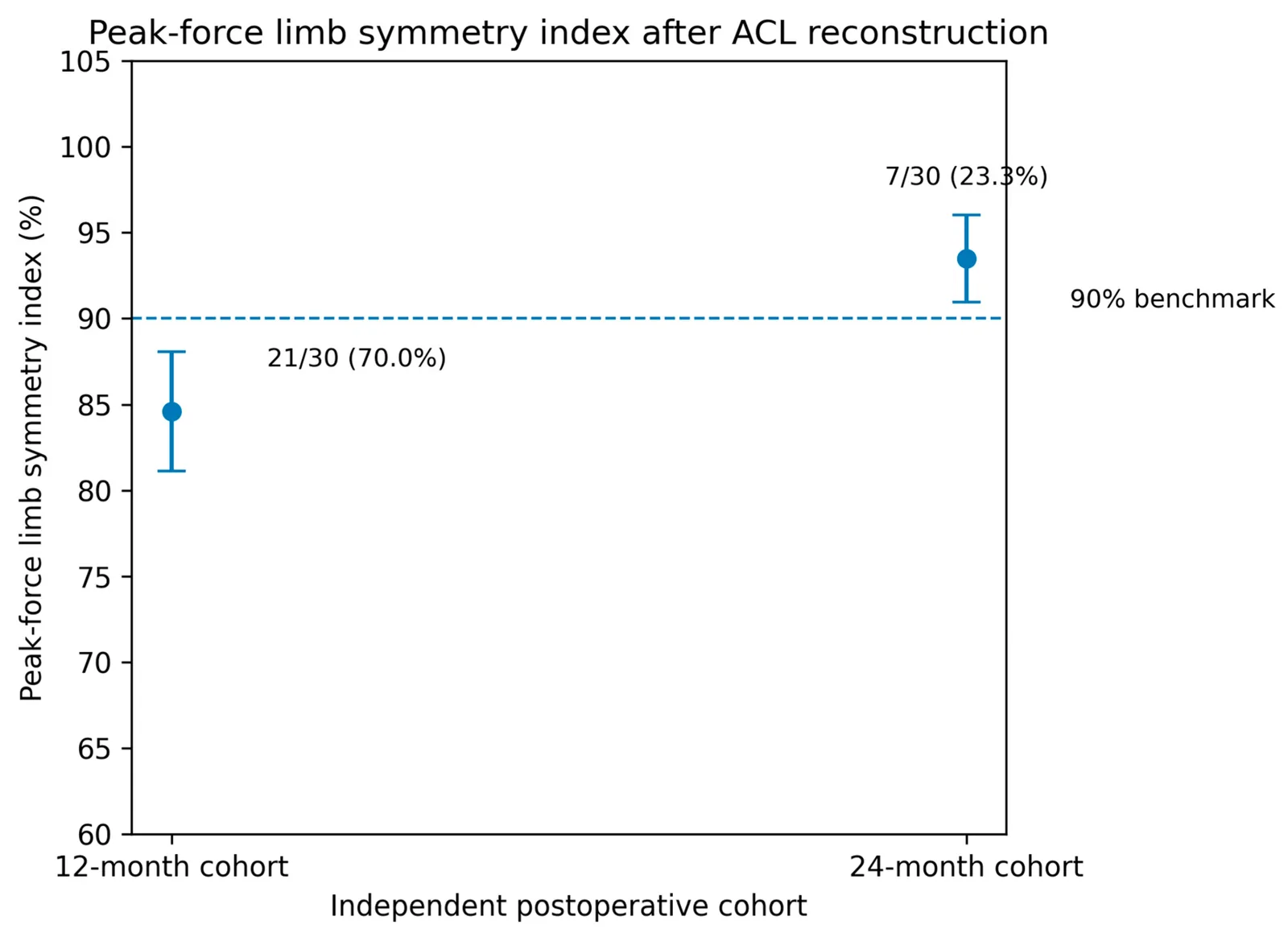
Peak-force limb symmetry index (PF LSI) in the independent 12- and 24-month postoperative cohorts. Points represent cohort means and error bars denote 95% confidence intervals for the cohort means. The horizontal dashed line indicates the descriptive 90% PF LSI benchmark. Text annotations report the proportion of athletes with PF LSI < 90% in each cohort.

**Figure 2:**
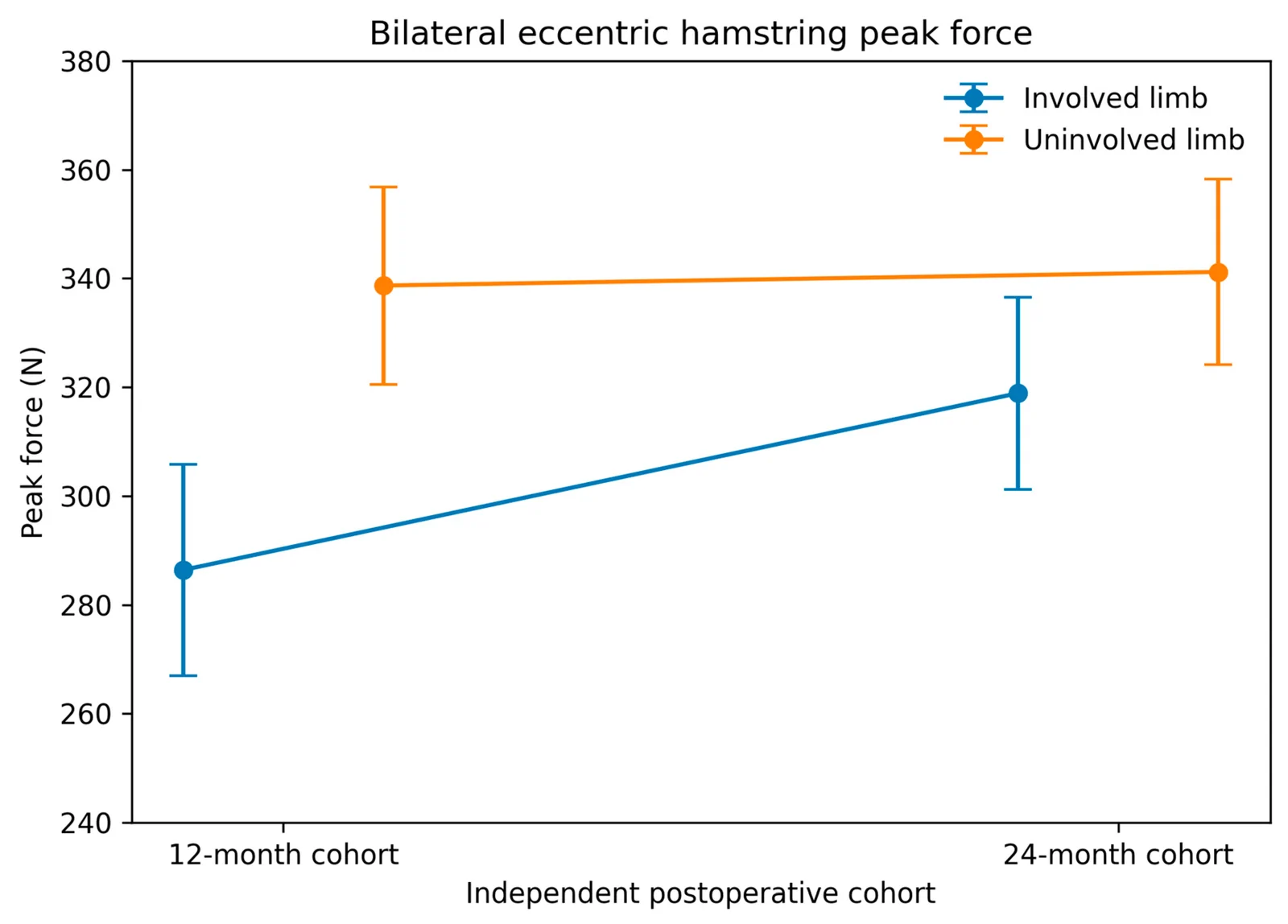
Bilateral eccentric hamstring peak force (PF) in the independent 12- and 24-month postoperative cohorts. Points represent cohort means and error bars denote 95% confidence intervals for the cohort means. The involved-limb PF differed between cohorts, whereas uninvolved-limb PF was similar.

Shapiro–Wilk testing did not indicate a violation of the normality assumption for PF LSI in either cohort or for the other continuous force outcomes that were analyzed parametrically. Between-cohort comparisons are summarized in Table 4. The primary PF LSI outcome is illustrated in Figure 1, and bilateral PF values are illustrated in Figure 2. The 24-month cohort had a higher PF LSI than the 12-month cohort (mean difference 8.9 percentage points, 95% CI 4.7 to 13.1; *p* < 0.001; d = 1.09). Involved-limb PF was 32.5 N higher in the 24-month cohort (95% CI 6.8 to 58.2; *p* = 0.014; d = 0.65), whereas uninvolved-limb PF differed by only 2.5 N (95% CI −21.9 to 26.9; *p* = 0.838; d = 0.05). These results indicate that the cross-sectional contrast in PF symmetry was driven predominantly by the involved limb rather than by a between-cohort difference in uninvolved-limb PF.

PF LSI was <90% in 21/30 athletes in the 12-month cohort (70.0%; 95% CI 52.1–83.3%) and 7/30 in the 24-month cohort (23.3%; 95% CI 11.8–40.9%). The between-cohort association was significant (Pearson χ^2^ = 13.13, df = 1, *p* < 0.001). Figure 1 summarizes PF LSI together with the descriptive 90% benchmark, while Figure 2 shows the involved- and uninvolved-limb PF values for each independent cohort.

## 4. Discussion

The main clinical finding was that the higher PF LSI in the independent 24-month cohort reflected higher involved-limb PF, whereas uninvolved-limb PF was similar between cohorts. Nevertheless, 7 of 30 athletes (23.3%) in the 24-month cohort remained below the descriptive 90% PF LSI benchmark. Clinically, a later postoperative stage does not guarantee normalized eccentric knee-flexor function, and late-stage bilateral testing may still identify residual deficits not apparent from time since surgery alone.

These findings extend, rather than replicate, the work of Högberg and colleagues. Their studies showed that NordBord-detected knee-flexor asymmetry can be present during the first postoperative year and remain detectable at two and five years after hamstring-autograft ACLR [8,24]. The present study adds a direct single-center comparison of two independent postoperative cohorts tested with the same protocol and reports bilateral PF values alongside PF LSI and the proportion below 90%.

The mechanism underlying this force pattern cannot be determined from these data. Hamstring tendon harvest is one plausible contributor because 70% of each cohort had a hamstring autograft and persistent musculotendinous changes after harvest are well described [12,13,14,15,17,18,19,20,21,22,23]. However, 30% of each cohort had a BPTB graft and graft-stratified force outcomes could not be reconstructed, so donor site morbidity cannot be treated as a universal explanation. Other plausible contributors include rehabilitation exposure, pain or effusion, intra-articular pathology, deconditioning, and altered neural activation; force loss alone does not diagnose AMI [3,4,29,30,31,32,33,39,40,41].

The clinical implication is not that a single NordBord threshold should determine RTS. LSI can overestimate recovery if the uninvolved limb is impaired, and contemporary RTS frameworks recommend integrating strength with symptoms, functional performance, movement quality, psychological readiness, and sport-specific exposure [5,6,28,42,43,44,45,46]. The 90% value remains a descriptive benchmark rather than a validated NordBord-specific clearance criterion.

No biological samples or structural donor site imaging were collected.

Table 5 is included as a literature-derived interpretive aid to contextualize an abnormal eccentric force finding; it is not a direct study outcome and does not assign a mechanism from NordBord data alone.

Future work should follow the same athletes longitudinally, retain participant-level trial data, stratify or adjust for graft type and activity level, and quantify rehabilitation exposure. Combining eccentric force testing with complementary isokinetic, imaging, and activation-specific measures would help determine why residual deficits in the involved limb remain in some athletes and whether they carry prognostic value for subsequent sport participation or reinjury.

## 5. Limitations

First, the 12- and 24-month groups were independent cross-sectional cohorts; therefore, the data describe between-cohort differences and not individual recovery trajectories.

Second, the retained records did not preserve the recruitment sequence or a prospective screening log. Consecutive versus convenience recruitment therefore cannot be confirmed, and the total number screened and detailed reasons for exclusion cannot be reconstructed, limiting assessment of the selection process.

Third, participant-level data were not available for the additional analyses undertaken during this revision. Covariate-adjusted analyses for Tegner Activity Score and graft-stratified force could therefore not be performed; the reported Tegner and uninvolved-limb PF comparisons were limited to calculations obtainable from the archived cohort summary statistics.

Fourth, hamstring tendon autograft was used in 70% of each cohort and BPTB in 30%. Although graft proportions were identical between cohorts, the absence of graft-stratified outcomes prevents determination of whether the same force pattern was present within each graft subgroup.

Fifth, the uninvolved limb is not necessarily a healthy normative reference after ACLR, and LSI may overestimate recovery when bilateral weakness is present [4,27]. No independent healthy control group was included.

Sixth, rehabilitation exposure was not standardized, and the study did not include preoperative strength, early postoperative baseline values, pain, effusion, knee laxity, quadriceps strength, or a standardized, objective RTS status. These unmeasured variables, including the modest between-cohort difference in Tegner score, may confound the unadjusted force comparisons.

Seventh, the study included only men and had a modest sample size. The benchmark power calculation indicates adequate sensitivity for a large standardized effect but does not rule out smaller clinically relevant differences and does not replace prospective sample-size planning.

Eighth, no structural imaging, direct voluntary-activation/neurophysiological reference standard, or blood/synovial molecular biomarker sampling were performed. NordBord outcomes are also task- and protocol-dependent [21,27]; the findings should therefore be interpreted as protocol-specific eccentric force differences rather than as a mechanism-specific diagnosis.

## 6. Conclusions

In this cross-sectional comparison of independent postoperative cohorts, the smaller eccentric hamstring asymmetry observed in the 24-month cohort was explained predominantly by higher involved-limb PF, whereas uninvolved-limb PF was similar between cohorts. Nevertheless, 23.3% of athletes in the 24-month cohort remained below the descriptive 90% PF LSI benchmark. The principal clinical message is therefore that postoperative time alone does not guarantee normalization of eccentric knee-flexor function; late-stage bilateral testing can identify residual involved-limb deficits and should be interpreted alongside absolute force, symptoms, graft history, rehabilitation exposure, and the broader RTS assessment rather than as a stand-alone diagnostic or clearance test.

## Figures and Tables

**Table 1:** Experimental protocol, operational definitions, and interpretative boundaries.

Component	Operational Detail	Unit/Calculation	Interpretive Role
Force acquisition	Simultaneous bilateral uniaxial force from the right and left ankle restraints during the Nordic hamstring exercise; NordBord sampling frequency 50 Hz.	N	Task-specific eccentric knee-flexor force measurement.
Examiner and standardization	All assessments were supervised by one trained examiner who was not involved in rehabilitation; the same protocol was used in both cohorts.	Same testing sequence	Reduces procedural variability between cohorts.
Warm-up and familiarization	5 min stationary cycling at 80–100 rpm; 3 submaximal Nordic repetitions; 2 min rest.	Standardized sequence	Preparation and familiarization before maximal testing.
Maximal testing	3 maximal Nordic hamstring trials with 1 min recovery. For PF, the highest value across trials was retained independently for each limb; retained limb values could originate from different trials.	Three trials	Eccentric force-testing phase.
Peak force (PF)	Highest force recorded after onset of the eccentric movement.	N	Primary maximal-force variable for each limb.
Mean force (MF)	Mean force recorded between movement onset and the PF event.	N	Secondary descriptor of force production across the eccentric phase.
Symmetry and bilateral force reporting	PF LSI and MF LSI = involved/uninvolved × 100; involved- and uninvolved-limb PF reported separately.	% and N	Quantifies relative symmetry while showing the limb-specific contribution to between-cohort differences.
PF LSI < 90%	Binary descriptive flag derived from PF LSI.	n (%)	Commonly used clinical benchmark; not a validated NordBord-specific RTS cut-off.
Diagnostic boundary	No structural imaging, direct voluntary-activation/neurophysiological reference standard, or molecular sampling was obtained.	Not applicable	Force findings identify functional impairment but cannot establish AMI, a specific donor site lesion, or a molecular diagnosis.

N = Newtons; AMI = arthrogenic muscle inhibition; LSI = limb symmetry index; MF = mean force; PF = peak force; RTS = return to sport.

**Table 2:** Participant characteristics.

Characteristic	12-Month Cohort (n = 30)	24-Month Cohort (n = 30)
Age, years	28.4 ± 5.1	29.1 ± 4.8
Height, cm	178.2 ± 6.3	179.0 ± 5.9
Body mass, kg	76.8 ± 8.4	77.3 ± 7.9
Time after ACLR, months	12.3 ± 0.8	24.1 ± 1.1
Hamstring tendon autograft, n (%)	21 (70)	21 (70)
BPTB autograft, n (%)	9 (30)	9 (30)
Tegner Activity Score	6.2 ± 1.1	6.8 ± 0.9

Values are presented as mean ± SD unless otherwise stated. ACLR = anterior cruciate ligament reconstruction; BPTB = bone–patellar tendon–bone.

**Table 3:** Descriptive bilateral eccentric hamstring force.

Outcome	Involved Limb	Uninvolved Limb	LSI, %
12 months: PF, N	286.4 ± 52.1	338.7 ± 48.6	84.6 ± 9.3
12 months: MF, N	241.8 ± 44.7	295.3 ± 42.1	81.9 ± 10.2
24 months: PF, N	318.9 ± 47.3	341.2 ± 45.8	93.5 ± 6.8
24 months: MF, N	274.6 ± 41.2	298.7 ± 39.6	91.9 ± 7.4

PF = peak force; MF = mean force; LSI = limb symmetry index (involved/uninvolved × 100).

**Table 4:** Between-cohort comparisons of eccentric hamstring outcomes.

Outcome	12 Months	24 Months	Mean Difference (24−12), 95% CI	Cohen’s D	*p* Value
PF involved, N	286.4 ± 52.1	318.9 ± 47.3	32.5 (6.8 to 58.2)	0.65	0.014
PF uninvolved, N	338.7 ± 48.6	341.2 ± 45.8	2.5 (−21.9 to 26.9)	0.05	0.838
PF LSI, %	84.6 ± 9.3	93.5 ± 6.8	8.9 (4.7 to 13.1)	1.09	<0.001
MF involved, N	241.8 ± 44.7	274.6 ± 41.2	32.8 (10.6 to 55.0)	0.76	0.005
MF LSI, %	81.9 ± 10.2	91.9 ± 7.4	10.0 (5.4 to 14.6)	1.12	<0.001

CI = confidence interval; PF = peak force; LSI = limb symmetry index.

**Table 5:** Differential diagnosis framework for interpreting an eccentric hamstring force deficit after ACLR.

Diagnostic Domain	Potential Explanation	Complementary Assessment	Interpretive Role
Functional capacity	Incomplete eccentric strength restoration; bilateral deconditioning.	Repeat standardized Nordic testing; bilateral PF; body-mass-normalized force and external normative data when available.	Confirms magnitude and reproducibility of the functional phenotype but not its cause.
Donor site/musculotendinous pathology	Semitendinosus/gracilis harvest; variable tendon regeneration; altered muscle morphology.	Graft/harvest history; posterior-thigh examination; MRI or musculoskeletal ultrasound when clinically indicated.	Can support a peripheral structural explanation for reduced force.
Neural activation	Reduced voluntary drive or arthrogenic inhibitory mechanisms.	Twitch interpolation or burst superimposition; appropriately designed sEMG; reflex or corticospinal measures.	Activation-specific testing is required before attributing force loss to AMI.
Joint pathology and symptoms	Pain, effusion, instability, or concomitant meniscal/chondral pathology.	Clinical examination; effusion and laxity assessment; symptom measures; imaging when indicated.	Identifies competing joint-related sources of force suppression or altered loading.
Rehabilitation and sport exposure	Insufficient high-force eccentric loading; deconditioning; incomplete return-to-training exposure.	Rehabilitation records; loading progression; strength-program dose; sport/training exposure.	Contextual and potentially modifiable contributor without implying tissue pathology.
Molecular joint milieu (research)	Inflammation and matrix turnover after ACL injury/reconstruction.	Synovial or serum cytokines; matrix metalloproteinases; COMP and other cartilage-turnover markers.	Exploratory biological phenotyping; no validated cut-off for hamstring weakness, AMI, or RTS.

AMI = arthrogenic muscle inhibition; COMP = cartilage oligomeric matrix protein; MRI = magnetic resonance imaging; PF = peak force; RTS = return to sport; sEMG = surface electromyography. Molecular assessments are presented as research tools rather than validated clinical tests for the observed force phenotype.

## Data Availability

The participant-level analytical dataset was not available to the authors for the current revision. The cohort-level summary data supporting the reported analyses are provided in the manuscript; no participant-level reanalysis was reconstructed from unavailable records.
